# Astragalus polysaccharide (APS) exerts protective effect against acute ischemic stroke (AIS) through enhancing M2 micoglia polarization by regulating adenosine triphosphate (ATP)/ purinergic receptor (P2X7R) axis

**DOI:** 10.1080/21655979.2021.1980176

**Published:** 2022-02-15

**Authors:** Xiang Jia, Liying Xie, Yuan Liu, Tianfu Liu, Peiqun Yang, Jianfang Hu, Zhichao Peng, Kangrui Luo, Min Du, Chaojun Chen

**Affiliations:** a Department of Neurology, Guangzhou Hospital of Integrated Traditional and West Medicine, Guangzhou, China; b Nursing Department, Guangzhou Hospital of Integrated Traditional and West Medicine, Guangzhou, China

**Keywords:** Acute ischemic stroke, astragalus polysaccharide, microglia, blood–brain barrier, purinergic receptor

## Abstract

Clinically, the effective treatment for patients with acute ischemic stroke (AIS) is very limited. Therefore, this paper aims to investigate the mechanism how astragalus polysaccharide (APS) exerts protective effect against AIS and provide a new method for the treatment of AIS. Cell surface antigen flow cytometry and immunofluorescence were used to identify M1 and M2 microglia. Western blot was used to evaluate the expression of associated protein. Oxygen-glucose deprivation (OGD) was used to simulate the effect of AIS on rat microglia. The middle cerebral artery occlusion (MCAO) model was established to simulate the effect of AIS *in vivo*. Evans blue dye (EBD) was used to evaluate the permeability of blood–brain barrier (BBB). Western blot and cell surface antigen flow cytometry results showed that APS promoted the M2 polarization of rat microglia by inhibiting the expression of purinergic receptor (P2X7R). APS reversed the effect of OGD on the polarization of rat microglia M1/ M2 by regulating P2X7R. APS reversed the effect of MCAO on the polarization of rat microglia M1/ M2 *in vivo*. Furthermore, APS inhibited the expression of P2X7R by promoting the degradation of adenosine triphosphate (ATP) in the cerebral cortex of MCAO rats. In addition, APS contributed to maintain the integrity of BBB. Summarily, APS can reduce brain injury by promoting the degradation of ATP in microglia and inhibiting the expression of P2X7R after AIS.

## Introduction

China is one of the countries with the highest incidence of stroke [1]. At present, the disease is the main cause of death in China, with an annual increase of 2.5 million cases and 1.6 million deaths due to stroke. Acute ischemic stroke (AIS) accounts for about 70% of stroke in China, and the mortality within one month is about 2.3–3.2% [2]. Therefore, effective prevention and treatment of AIS is of great significance to patients and their families.

Studies have shown that inflammation/ immune response runs through the whole process of AIS. The main causes of nerve injury after AIS are the failure of neurons to complete mitochondrial aerobic respiration, the decrease of intracellular pH value, the change in ion gradient of cell membrane and cytotoxic edema caused by apoptosis swelling [3]. Damaged neurons and glial cells release high levels of adenosine triphosphate (ATP), activate purinergic receptor (P2X7R), and release a large number of inflammatory mediators to induce neuroimmune disorders and inflammation [4]. Currently, the treatment of AIS mainly focused on reflow and brain protection, and the clinical problem have not been solved: intravenous thrombolysis and mechanical thrombectomy within time window can only solve the cerebral vascular reflow of very few patients and cannot interfere with cascade events of brain injury events, the treatment of most patients still depends on brain protection measures [5]. Unfortunately, there are still few effective neuroprotective measures in clinic, which highlights the importance of targeted inflammatory/ immune interventions in current neuroprotection studies after AIS.

At present, pharmacological studies have found that astragalus polysaccharide (APS) has a wide range of immune regulation, anti-tumor, anti-oxidation, antihypertensive, hypoglycemic, liver and kidney protection, and has broad application prospects in anti-atherosclerosis (AS) disease [6]. Inflammatory mediators play an important role in the progression of ischemic penumbra injury. By reducing the neuro-inflammatory response, neuroprotection after stroke can be achieved [7], and microglia is the most effective regulatory target for brain repair and regeneration [8]. Under different cell microenvironment conditions, microglia can differentiate into two phenotypes, namely M1 type with pro-inflammatory effects and M2 type with anti-inflammatory effect [9]. In the early stage after ischemic injury, the locally activated microglia exhibited M2 phenotype, but M2 type polarization response was transient, which was replaced by an inflammation and harmful reaction dominated by M1 polarization cells within a few days after ischemic injury. The phagocytosis and release of neurotoxic mediators, such as TNF (tumor necrosis factor), IL-1 (interleukin-1), IL-6, McP-1 (monocyte chemotactic protein-1), MIP-1 (macrophage inflammatory protein-1), ROS (reactive oxygen species), NO (nitric oxide), matrix, and MMPs (metalloproteinases), were decreased in M1 polarized cells. In the later stage, in order to fight against this inflammatory and harmful process, damaged neurons in the penumbra area produce IL-4, an effective M2 polarization promoter [10]. At present, the difference between M1 and M2 microglia depends on their respective characteristic surface markers, M1 markers: HLA-DR (human leukocyte antigen), CD16 (cluster of differentiation), CD32, CD86, etc., and M2 surface markers: CD209, CD206, CD301, CD163, Arg-1, and Ym-1 [11,12]. Studies have found that antibacterial drugs (minocycline and azithromycin), eplerenone and spironolactone [13,14], metformin [15], rosiglitazone [6], etc., which can't reduce the M1/M2 ratio after AIS [16-18], play a protective role in the brain. This suggests that inhibiting the differentiation of microglia M1 and promoting the differentiation of M2 to achieve neuroprotective effect could be a new strategy for the treatment of AIS. However, the effect of APS on M2 microglia polarization remains unknown.

In AIS, blood–brain barrier (BBB) damage leads to neurological dysfunction [19]. Therefore, the preservation of BBB integrity could ameliorate AIS-induced brain injury [20,21]. Moreover, numerous studies have indicated that M2 microglia polarization plays a protective role in BBB integrity [22,23]. Whereas, the role of APS in BBB integrity is not clear.

This study aimed to investigate the mechanism how APS exerts protective effect against AIS and verify whether APS enhanced the M2 polarization of microglia through suppressing ATP-mediated P2X7R expression to maintain the integrity of BBB. Results of the present study would provide a new method for the treatment of AIS.

## Materials and methods

### Ethnics statement

All mice were placed in a pathogen-free environment of Model Animal Research Center of Nanjing University. All protocols were approved by the Institutional Committee for Animal Care and Use at Model Animal Research Center of Nanjing University. All animal works were carried out in accordance with the approved protocol.

### Reagent

Penicillin, fetal bovine serum (FBS), DMEM/ F12 medium, and PBS were purchased from GE™ Hyclone. APS was purchased from Beijing Solarbio Science & Technology Co., Ltd. ATP was purchased from Shanghai Zeye Biological Technology Co., Ltd. Rat ATP ELISA Kit and rat ADP ELISA Kit were purchased from Beijing winter song Boye Biotechnology Co. Ltd. EBD was purchased from Real-Times (Beijing) Biotechnology Co., Ltd. EDTA antigen repair solution was purchased from Servicebio. Antibodies: anti-GAPDH (KC-5G5, KangChen Bio-tech), anti-P2X7R (BA2808, Boster Biological Technology Co., Ltd.), anti-CD163 (ab182422, Abcam), anti-CD206 (sc-58,986, Santa Cruz), anti-CD86 (NBP2-25,208, Novus), anti-HLA-DR (MA5-32,232, Invitrogen), anti-CD163-PE (85–12-1639-41, eBioscience), anti-CD206-APC (85–17-2069-41, eBioscience), anti-CD86-PE (70-AR08604-100, MultiSciences), and anti-HLA-DR-APC (70-AH0HD05-20, MultiSciences).

### Cell culture

HAPI cells were cultured in DMEM/ F12 medium containing 10% FBS (SH30087.01, Hyclone), 100 U/ml penicillin (SH30010, Hyclone), and 100 mg/ml streptomycin in a humidified atmosphere at 37°C with 5% CO_2_.

### Western blot

Western blot was used to detect target proteins extracted from HAPI cells. Whole cell lysates were extracted with lysis buffer: 50 mM Tris pH7.4, 150 mM NaCl, 1 mM EDTA, 1% Triton, and 10% glycerol and a mixture of protease and phosphatase inhibitor cocktail (Roche, Basel, Switzerland), protein concentrations were determined by the Bradford assay. Soluble protein (30–40 μg) was subjected to SDS-polyacrylamide gel electrophoresis. Separated proteins were electrophoretically transferred to polyvinylidene difluoride (PVDF) membranes (Millipore, Billerica, MA, USA). Primary antibody used in this study was diluted into 5% nonfat milk at a ratio of 1: 500 [24].

### Cell surface antigen flow cytometry

HAPI cells in logarithmic growth phase were seeded on a 6-well plate with a density of 2 × 10^5^/ well. They were treated with PBS (0.01 mol/ L), ATP (3 mmol/ L) or ATP (3 mmol/ L) for 24 h respectively, and then APS (100 mg/ L) for 48 h. The cells in each group were collected, digested with 0.05% trypsin, and then resuspended into single cell suspension. The cells were washed twice with PBS (centrifugation at 800 rpm for 5 min), and the cell concentration was adjusted to 1 × 10^6^ cells/ml with medium. 500 μl cell suspension was added into each measuring tube, and then PE or APC labeled HLA-DR (1.5 μg/ mL), CD86 (50 μg/ mL), CD206 (12 μg/ mL), and CD163 antibodies (50 μg/ mL) were added. The cells were then washed twice with PBS (centrifuged at 800 rpm for 5 min). Flow cytometry was used to obtain the results. Besides, a logical gating strategy was applied. The M1 microglia cell subpopulation refers to dot plot HLA-DR versus CD86, while the M2 microglia cell subpopulation refers to CD163 versus CD206.

### Oxygen-glucose deprivation (OGD)

HAPI cells in logarithmic growth phase were seeded on a 6-well plate with a density of 2 × 10^5^/ well, then cultured in a sugar-free and serum-free DMEM medium at 37°C in 95% N_2_ and 5% CO_2_ for 6 hours, and then cultured at 37°C under normal condition (95% O_2_, 5% CO_2,_ DMEM medium containing 10% FBS) for 72 h before collecting samples for detection.

### In vitro *model of brain–blood barrier (BBB)*

The inserter was precoated with 2% gelatin. Next, HAPI cells were evenly seeded in a 24-well cell inserter with a density of 200,000 cells/cm^2^ and culture in the CO2 incubator (5% CO_2_, saturated humidity, 37℃) until confluence. After observing the confluence of HAPI cells under the microscope, the cell culture medium was added into the donor pool of inserter to make the liquid level difference between the donor pool and the recipient pool >0.5 cm. Subsequently, leakage test was utilized to identify whether BBB was established. If there was still obvious liquid level difference between the two cisterns of the inserter after 4 h, it was considered that the HAPI cells had been completely converged and BBB was established.

### Detection of the permeability of APS through the BBB

The permeability of APS through the BBB was identified by detecting the APS level in culture medium of the recipient pool using liquid chromatograph-mass spectrometer (LC-MS). The chromatographic conditions were listed as follow: chromatographic column, Phenomenex kinetex C18 (100 mm × 2.1 mm, 1.7 μm) ; column temperature, 30℃; injection volume, 10 μL; flow rate, 0.4 mL/min; mobile phase, methanol (a) and water (b). Besides, gradient elution procedure was performed as follow: 0 ~ 0.5 min, 10% A; 0.5 ~ 1.8 min, 10%~90% A; 1.8 ~ 3.8 min, 90%A;3.8 ~ 4.5 min, 90%~5% A; 4.5 ~ 6.0 min, 5% A. In addition, mass spectrum conditions were listed as follow: ion source, electrospray ionization (ESI+); scanning mode, positive ion scanning mode; ion source temperature, 120°C; capillary voltage, 3.0 kV; desolvent gas temperature, 500°C; desolvent gas flow, 850 L/hr; collision gas flow rate, 0.11 mL/min; conical hole back blowing gas flow, 150 L/hr; detection mode, multi reaction monitoring (MRM) mode.

### Construction and Longa score of MCAO rat model

Male SD rats aged 4–6 weeks (Model Animal Research Center of Nanjing University), were fed adaptively for 1 week. The rats were divided into five groups with three rats in each group, which were labeled as control group, MCAO group, MCAO+ normal saline group, MCAO+APS low-dose group, and MCAO+APS high-dose group. The standard MCAO model construction steps [25] were followed to operate and refer to the Longa scoring method for neurobehavioral scoring. Scoring criteria: normal, no neurological deficit, 0 point; when lifting tail, contralateral forelimb of the brain lesion cannot be fully extended, mild neurological deficit, 1 point; when walking on the ground, the rats turned to the contralateral side of the brain lesion and had moderate nerve function defect, 2 points; while walking on the ground, the rat’s body falls to the opposite side of the brain lesions and had severe neurological deficits, 3 points; unable to walk independently and have consciousness loss, 4 points. The success criteria of modeling: neurobehavioral score 1–3, 0, and 4 were eliminated. Longa score are shown in Table 1.

**Table 1.** Longa score

score	Number of rats
0	1
1	2
2	9
3	5
4	0

A total of 20 rat models, one died of asphyxia due to accidental injury of vagus nerve. Two bleeding rats were removed. 17 models were completed. According to Longa scoring method, the success rate of the model was 94.1%, and the success rate = number of successful models/total number of models× 100%.

### Administration

After MCAO rats were successful established, the rats were intraperitoneally injected with APS (low-dose group: 22.5 mg/kg, high-dose group: 45 mg/kg), The control group was given an equal volume of normal saline, once a day, according to groups, they were administered for 1 day, 3 days, or 5 days. After the administration, the rats were decapitated, and the brain tissue and serum were collected for subsequent experimental detection.

### ELISA detection

SD rat cortical brain tissues of control group, MCAO group, MCAO+normal saline group, MCAO+APS low-dose group, and MCAO+APS high-dose group were collected and preserved. After the sample is cut, it is weighed, frozen rapidly with liquid nitrogen, and homogenized fully with homogenizer. Add appropriate amount of PBS, centrifuge for 20 min (2000–3000 rpm), and carefully collect the supernatant. Rat ATP ELISA KIT (DG20151D, Beijing Dongge Biology) and rat ADP ELISA KIT (DG20962, Beijing Dongge Biology) were used for detection. The absorbance (OD value) of each well was measured at the wavelength of 450 nm.

### Immunofluorescence

Paraffin sections were dewaxed sequentially in the order of 15 min for xylen I, 15 min for xylen II, 5 min for absolute ethanol I, 5 min for absolute ethanol II, 5 min for 90% alcohol, 5 min for 80% alcohol, and 5 min for 70% alcohol. Finally, the sections were washed with distilled water. After the sections were repaired with EDTA antigen recovery solution, they were incubated in 5% BSA for 30 min at room temperature. Gently shaked off the blocking solution, added anti-CD163 (ab182422, Abcam, 1:100), anti-CD206 (sc-58,986, Santa Cruz, 1:50), anti-CD86 (NBP2-25,208, Novus, 1:100), anti-HLA-DR (MA5-32,232, Invitrogen, 1:100). Then incubated overnight in a wet box at 4°C. The slices were placed in PBS (pH 7.4) and washed with decolorizing shaker for 3 times, 5 min each time. After the slices were slightly dried, the secondary antibody (dilution ratio: 1:200) corresponding to the primary antibody was dropped into the circle to cover the tissue, and incubated in the dark at room temperature for 50 min. DAPI counter-stained nuclei: the slices were placed in PBS (pH 7.4) and washed with decolorizing shaker for 3 times, 5 min each time. After the slices were shaken dried, DAPI dye solution was added dropwise to the circle, and incubated in the dark for 10 min at room temperature. Sealed the film and took pictures under the microscope.

### Detection blood–brain barrier (BBB) integrity in rat by Evans Blue Dye (EBD)

EBD (EB001, Real-Times (Beijing) Biotechnology Co., Ltd.) was injected intravenously 2 h before death. Normal saline was perfused into the heart to remove the residual blood in the cerebral vessels. The brain tissue was quickly taken out, weighed, then placed in 37°C formamide (1 ml/ 100 mg) for 48 h. After centrifugation, the supernatant was taken out and the absorbance of the supernatant was measured at 620 nm by spectrophotometer. According to the standard curve, the absorbance value was converted into EBD content to evaluate the permeability of BBB [26,27].

### Statistical analysis

In this study, all experiments were repeated at least twice, and average value of the three experiments was presented by the mean standard deviation (SD) calculated by STDEV formula in Excel. Shapiro–Wilk test was utilized to assess data distribution while data that do not exhibit a normal distribution was analyzed via rank sum test. The significance of all data was estimated by a Tukey’s multiple-comparison test in the ANOVA analysis using the SigmaStat 3.5 software. Statistical significance was accepted when *P*< 0.05.

## Results

This paper aims to investigate the mechanism how APS exerts protective effect against AIS and provide a new method for the treatment of AIS. We hypothesized that APS enhanced the M2 polarization of microglia through suppressing ATP-mediated P2X7R expression to maintain the integrity of BBB.

### APS promotes the M2 polarization of rat microglia by inhibiting P2X7R expression

**Figure 1.**
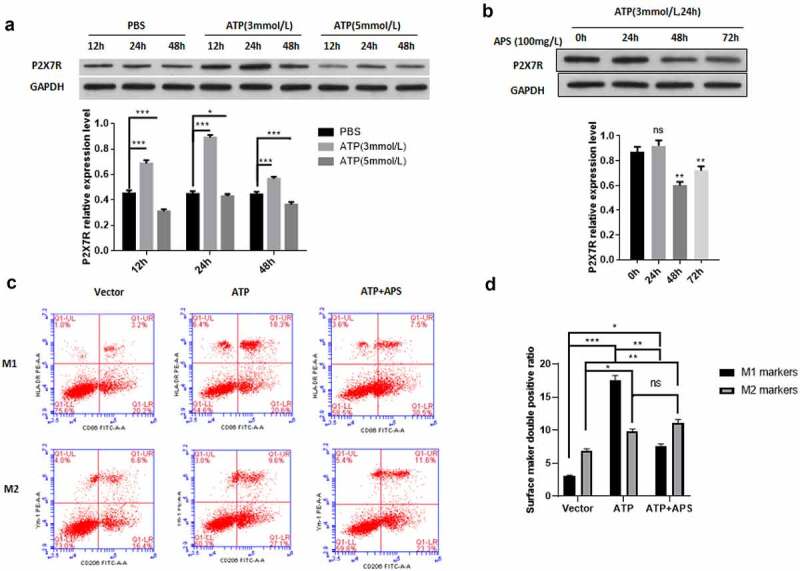
APS promotes M2 polarization of rat microglia by inhibiting P2X7R expression A: Western blot analysis of P2X7R expression under ATP stimulation (upper) and statistical analysis of P2X7R expression level based on Western blot results (lower), indicated antibodies were added during Western blot; **B**: Western blot analysis of P2X7R expression under ATP stimulation and APS treatment (upper) and statistical analysis of P2X7R expression levels based on Western blot results (lower), indicated antibodies were added during Western blot; **C**: Cell surface antigen flow cytometry of M1 and M2 microglia under indicated conditions, indicated antibodies were added during flow cytometry; **D**: Statistical analysis of the cell surface antigen flow cytometry. Data were representative of three independent experiments and analyzed by unpaired t-test. The error bars indicate SD. *P < 0.05.

After AIS, the damaged neurons and glial cells can release high concentration of ATP, which can activate P2X7R, release a large number of inflammatory mediators, and induce neuroimmune disorder and inflammation [4]. Then, we used different concentrations of ATP to detect the expression of P2X7R in rat microglia cell line HAPI at different time. As shown in Figure 1(a), compared with the control group, ATP significantly up-regulated the expression level of P2X7R in HAPI cells. In addition, treatment with 3 mmol/ L ATP for 24 h had the most significant effect on the expression level of P2X7R (Figure 1(a)). This treatment condition was used in subsequent experiments. Then we examined the effect of APS on P2X7R expression under ATP stimulation. We found that APS significantly reduced the increase of P2X7R expression induced by ATP stimulation (Figure 1(b)). Previous studies have shown that P2X7R promotes the activation of M1 microglia [28,29]. Therefore, we tested the effect of APS on M1/ M2 polarization of HAPI cells. The results showed that APS significantly inhibited the promoting effect of ATP on HAPI M1 polarization, thereby increasing the proportion of HAPI M2 polarization (Figure 1(c-d)). Taken together, these results indicated APS promotes the M2 polarization of rat microglia by inhibiting the expression of P2X7R.

### APS reverses the effect of OGD on rat microglia M1/M2 polarization by regulating P2X7R

**Figure 2.**
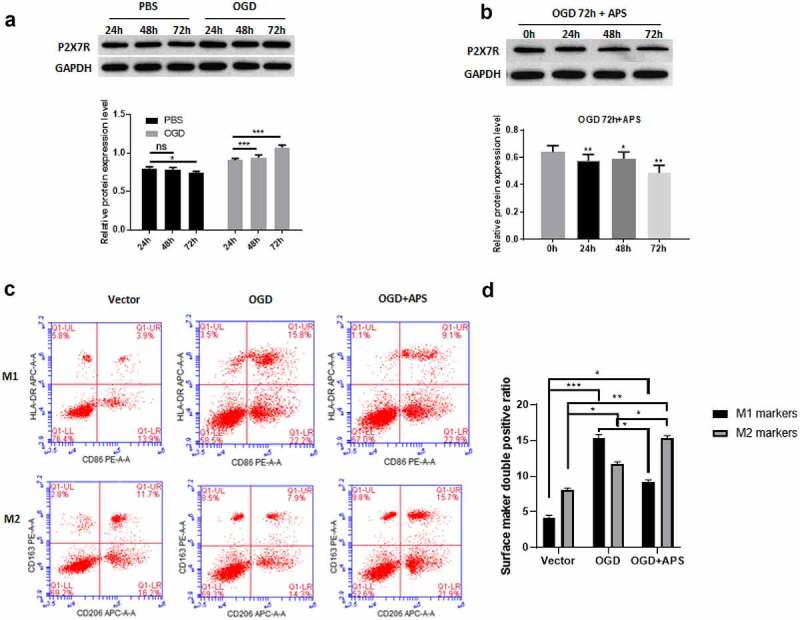
APS reverses the effect of OGD on the rat microglia M1/M2 polarization by regulating P2X7R A: Western blot analysis of P2X7R expression under OGD treatment (upper) and statistical analysis of the P2X7R expression levels based on Western blot results (lower), indicated antibodies were added during Western blot; B: Western blot analysis of P2X7R expression under OGD treatment and APS treatment (upper) and statistical analysis of P2X7R expression levels based on Western blot results (lower), indicated antibodies were added during Western blot; C: Cell surface antigen flow cytometry of M1 and M2 microglia under indicated conditions, indicated antibodies were added during flow cytometry; D: Statistical analysis of the cell surface antigen determined by flow cytometry. Data were representative of three independent experiments and analyzed by unpaired t-test. The error bars indicate SD. *P < 0.05.

We use HAPI cells OGD model to simulate the effect of AIS on rat microglia. Western blot results showed that compared with the control group, OGD treatment significantly increased the expression level of P2X7R in HAPI cells (Figure 2(a)). Furthermore, OGD treatment for 72 h had the most significant effect on the expression level of P2X7R (Figure 2(a)). This treatment condition was used in subsequent experiments. We found that APS significantly reduced the increase of P2X7R expression induced by OGD treatment (Figure 2(b)). In addition, OGD treatment significantly promoted M1 polarization of HAPI cells, which was similar to the effect of ATP stimulation on M1/M2 polarization of HAPI cells (Figure 2(c-d)). However, the effect of OGD treatment on HAPI M1 polarization were reversed by APS, which significantly increased the proportion of HAPI M2 polarization (Figure 2(c-d)). These data suggested that APS reversed the effect of OGD treatment on the M1/M2 polarization of rat microglia by regulating P2X7R.

### APS ameliorates ATP or OGD-repaired BBB integrity

**Figure 3.**
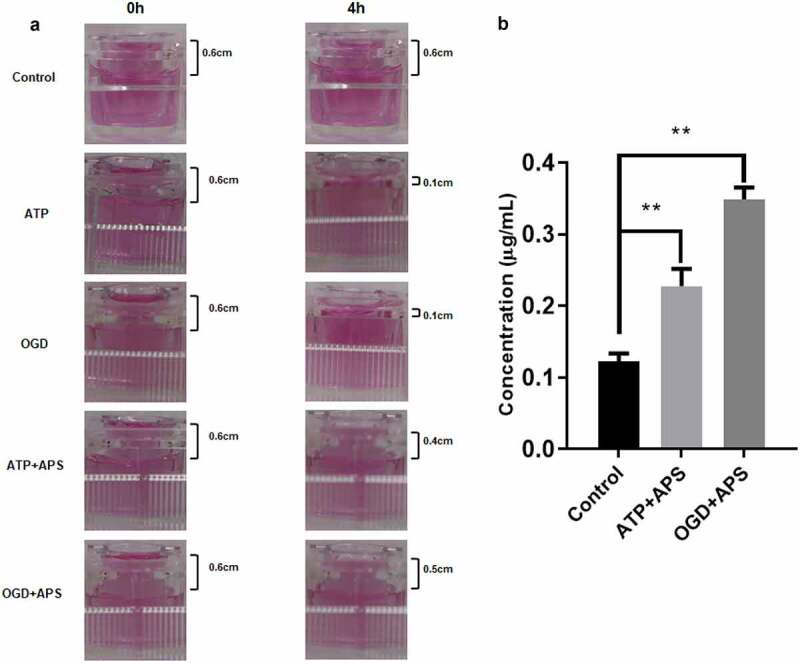
APS ameliorates ATP or OGD-repaired BBB integrity A: Images of the liquid level difference between the two cisterns of the inserter detected by leakage test in Control group, ATP group, OGD group, ATP+APS group and OGD+APS group. **B**: The APS level in culture medium of the recipient pool detected by LC-MS in Control group, ATP+APS group and OGD+APS group. **P < 0.01.

To further identify the effect of APS on ATP or OGD-repaired BBB integrity, leakage test was used to evaluate the permeability of *in vitro* model of BBB. Results showed that there was still obvious liquid level difference between the two cisterns of the inserter after 4 h in the control group, suggesting that *in vitro* model of BBB was established (Figure 3(a)). Besides, both ATP treatment and OGD reduces liquid level difference between the two cisterns of the inserter (Figure 3(a)), suggesting that ATP treatment and OGD might promote the permeability of *in vitro* model of BBB by repairing integrity. However, APS abolished the effects of ATP treatment and OGD on the permeability of *in vitro* model of BBB (Figure 3(a)). In addition, results of LC-MS indicated that APS could permeate through the *in vitro* model of BBB (Figure 3(b)). Therefore, these results suggested that APS could attenuate ATP or OGD-repaired BBB integrity.

### APS inhibits P2X7R expression by promoting ATP degradation in the cerebral cortex of MCAO rats

**Figure 4.**
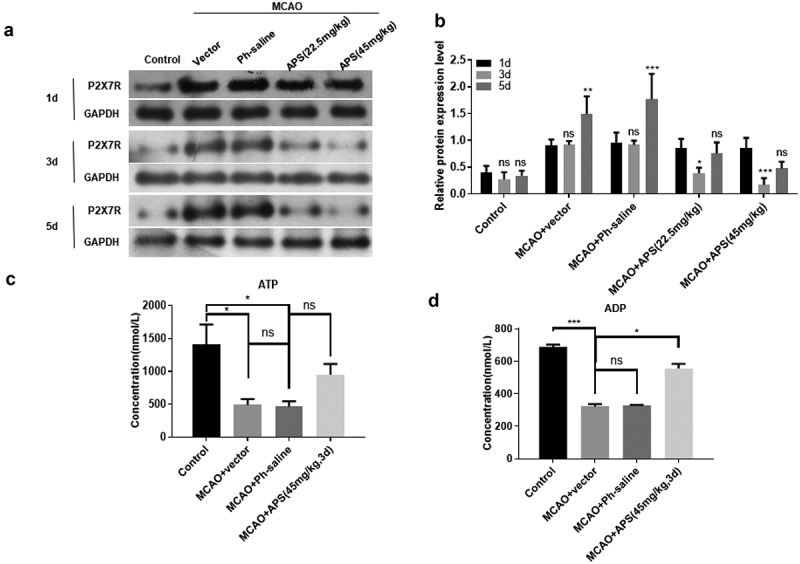
APS inhibits P2X7R expression by promoting ATP degradation in cerebral cortex of MCAO rats A: Western blot analysis of P2X7R expression in cortex of rats in control group, MCAO group, MCAO+normal saline group, MCAO+APS low-dose group or MCAO+APS high-dose group, indicated antibodies were added in the Western blot; **B**: Statistical analysis of the expression level of P2X7R based on Western blot results; **C**: ATP concentration of rat cortex of control group, MCAO group, MCAO+ normal saline group, MCAO+APS low-dose group or MCAO+APS high-dose group was detected by ELISA; **D**: ADP concentration of rat cortex of control group, MCAO group, MCAO+normal saline group, MCAO+APS low-dose group or MCAO+APS high-dose group was detected by ELISA. Data were representative of three independent experiments, and analyzed by unpaired t-test. The error bars indicate SD. *P < 0.05, **P < 0.01.

The MCAO model of SD male rats was used to simulate the effect of AIS *in vivo*. As shown in Figure 4(a), compared with the control group, the expression level of P2X7R in the cortex of MCAO model rats was significantly increased. APS significantly reversed the increase of P2X7R expression in cerebral cortex of MCAO model rats (Figure 4 (a-b)). In addition, 45 mg/kg APS treatment for 3 days had the most significant effect on the expression level of P2X7R (Figure 4(a-b)). This treatment condition was used in subsequent experiments. ELISA results showed that APS significantly reduced the ATP concentration in the cortex of MCAO model rats (Figure 4(c)). Meanwhile, APS significantly increased the concentration of ADP in the cortex of MCAO model rats (Figure 4(d)). Taken together, these data indicated that APS inhibited the expression of P2X7R by promoting the degradation of ATP in the cerebral cortex of MCAO model rats.

### *APS reverses the effect of MCAO on M1/ M2 polarization of rat microglia* in vivo

**Figure 5.**
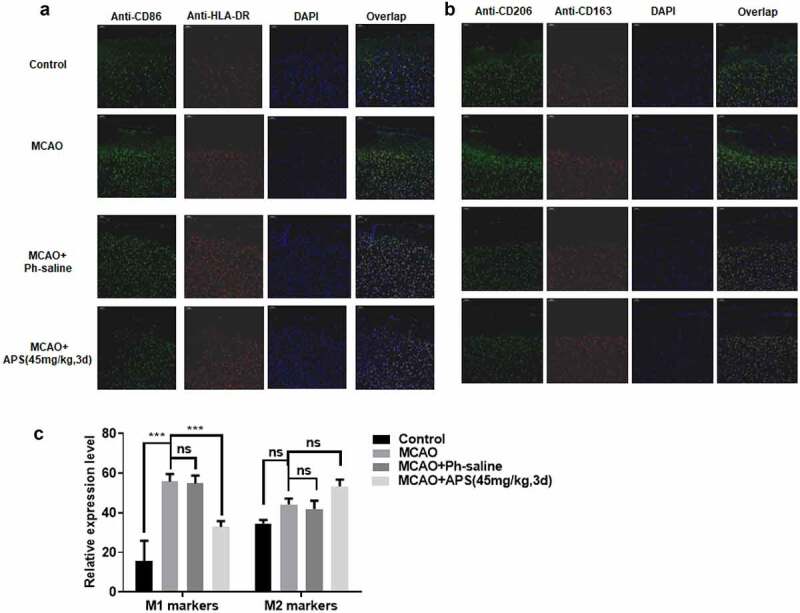
APS reverses the effect of MCAO on rat microglia M1/M2 polarization *in vivo* A&B: Immunofluorescence assay of M1/M2 markers in cortex of rats in control group, MCAO group, MCAO+normal saline group, MCAO+APS low-dose group or MCAO+APS high-dose group, indicated antibodies were added during immunofluorescence assay; C: Statistical analysis of the immunofluorescence assay. Data were representative of three independent experiments, and analyzed by unpaired t-test. The error bars indicate SD. *P < 0.05.

Immunofluorescent analysis was performed on paraffin sections of cerebral cortex of MCAO model rats under different treatment conditions. We used specific cell surface markers to distinguish M1 microglia from M2 microglia. As shown in Figure 5a, MCAO treatment significantly promoted the polarization of M1 rat microglia, and APS reversed the promotion effect of MCAO treatment. In addition, APS treatment significantly increased the proportion of M2 microglia in MCAO model rats (Figure 5(b)). In general, MCAO treatment significantly increased the polarization of M1 microglia, and APS reversed the effect. Furthermore, APS treatment promoted the polarization of M2 microglia, thus increasing the proportion of M2 microglia (Figure 5(c)).

### APS contributes to maintain the integrity of BBB

**Figure 6.**
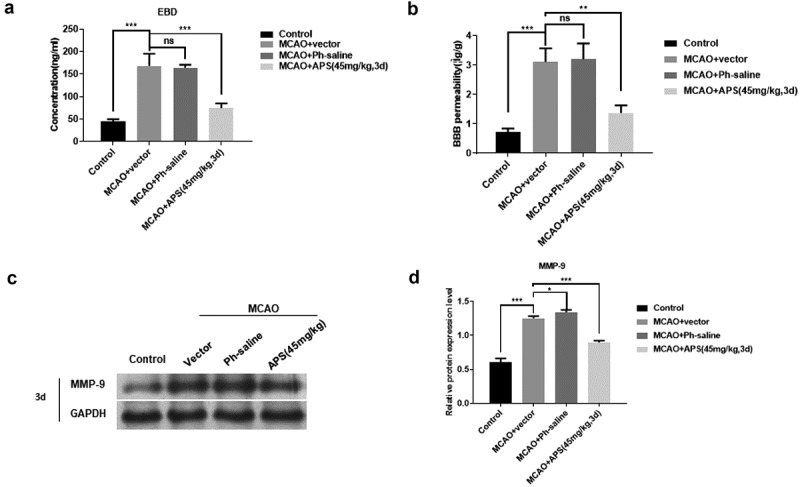
APS contributes to maintain the integrity of BBB A&B: EBD of cortex of rat in control group, MCAO group, MCAO+normal saline group, MCAO+APS low-dose group or MCAO+APS high-dose group; **C**: Western blot analysis of MMP-9 expression of rat cortex in control group, MCAO group, MCAO+normal saline group, MCAO+APS low-dose group or MCAO+APS high-dose group, indicated antibodies were added during Western blot; **D**: Statistical analysis of P2X7R expression levels based on Western blot results. Data were representative of three independent experiments, and analyzed by unpaired t-test. The error bars indicate SD. *P < 0.05; **P< 0.01; ***P< 0.001.

P2X7R is activated after AIS and releases pro-inflammatory mediators, such as TNF-α, IL-1β, ROS, MMPs, etc. [30,31]. These mediators promote the recruitment of white blood cells and degrades the extracellular matrix, resulting in the destruction of the BBB [32,33]. Therefore, we speculate that APS might participate in maintaining the integrity of BBB. To verify this hypothesis, we performed EBD to determine the integrity of BBB *in vivo*. EBD data showed that compared with the control group BBB permeability level of MCAO model rats was significantly increased, which was reversed by APS (Figure 6(a-b)). As shown in Figure 6(c-d), the expression level of MMP-9 protein in MCAO model rats was significantly higher than that in the control group and APS reversed the increase on MMP-9 protein expression. Taken together, these data indicated that APS maintained the integrity of BBB by inhibiting the expression of MMPs protein.

## Discussion

Despite the clinical treatment of AIS has made great progress in recent years, there is still a lack of effective treatment for AIS [34-37]. The difficulty of AIS treatment is due to our limited understanding of the mechanism of brain protection after AIS at the molecular level. Thus, it is of great significance to elucidate the targeted inflammation/immune interventions in neuroprotection after AIS [34,38]. Here, we reported the mechanism of APS promoting M2 polarization of rat microglia, which could be a therapeutic opportunity for AIS patients.

Traditional Chinese Medicine (TCM) has a history of thousands of years. It has accumulated rich experience and information in the prevention and treatment of stroke and has played an active and important role in the treatment of stroke. ‘Buyang Huanwu decoction’ is a classic treatment of stroke in TCM. Its clinical effect is reliable and has been widely accepted by the industry [39,40]. The characteristic of this prescription is that APS is the absolute main drug (original prescription composition: APS 120 g, Angelica 6 g, Red peony 5 g, Earthworm, Ligusticum chuanxiong, Safflower and Peach kernel 3 g, Astragalus accounted for 84%), and there is the therapeutic effect was in a dose effect relationship with the amount of astragalus [39,40]. These suggests APS plays an important role in clinical treatment of AIS, which is consistent with our finding that APS promotes the M2 polarization of rat microglia by inhibiting the expression of P2X7R.

Studies have found that in the process of inflammatory response, high extracellular ATP mediated inflammatory cells participate in the immune response mainly due to the over activation of P2X7R [41], and ATP is also the only natural agonist of P2X7R, which is consistent with our findings that APS inhibited P2X7R expression by promoting the degradation of ATP in cerebral cortex of MCAO model rats. Studies have found that antibacterial drugs (minocycline and azithromycin) can reduce the ratio of M1/ M2 after AIS, indicating that inhibiting the differentiation of microglia M1, promoting the differentiation of M2 and achieving neuroprotection may be a new strategy for the treatment of AIS in the future. And our data demonstrated that APS could promote M2 polarization of rat microglia and decreased M1/M2 ratio after AIS, both *in vitro* and *vivo* model. In addition, APS also up-regulated the expression of CD36, IL-12, and IL-27 on the surface of DCs membrane and down regulated the expression of IFI16, indicating that APS promotes the maturation and differentiation of DCs and has a positive intervention effect on the occurrence and development of AS [6].

The main component of basement membrane is extracellular matrix (ECM) molecules (main substances are IV collagen and laminin), which is an important part of BBB and maintains the integrity of BBB [42-46]. MMPs are the main degradation enzymes of ECM, which are closely related to the destruction and reconstruction of ECM in vascular wall [43,47]. In addition, P2X7R, activated by AIS, releases proinflammatory mediators, such as TNF-α, IL-1β, ROS, MMPs, etc. [30,31]. Our data show that APS reverses the increase of MMP-9 protein expression induced by MCAO by inhibiting P2X7R expression. Furthermore, EBD data showed that APS maintained the integrity of BBB by inhibiting the expression of MMPs protein. These results suggested the importance of APS in maintaining the integrity of BBB and clinical of treatment of AIS.

## Conclusions

Our current study demonstrated the APS function of promoting the M2 polarization of rat microglia and reducing the ratio of M1/M2 after AIS. Given the M1/M2 ratio after AIS is closely related to neuroprotection, our current work may offer a therapeutic opportunity for AIS patients.

## Highlights

- APS enhances AIS-suppressed M2 microglia polarization.
- APS inhibits P2X7R expression by promoting the degradation of ATP to M2 microglia polarization.
- APS ameliorates AIS-repaired BBB integrity through promoting M2 microglia polarization.
